# Supplementary material for: Chromothripsis during telomere crisis is independent of NHEJ, and consistent with a replicative origin
Source: Genome Res. 2019 May;29(5):737–49. doi: 10.1101/gr.240705.118 (PMC6499312; doi:10.1101/gr.240705.118)
Supplement: Supplemental Material [file supp_gr.240705.118_Supplemental_file_1.zip › contigs/annotated_contigs/DB39/contig.2.DB39_length_576_mean_cov_4.42708333333.docx]

**DB39_length_576_mean_cov_4.42708333333**

```
CAGCCATAAAAAGAATGAAATCATGTCGTTTGCAGAAAAATGGATGCATCTGGAGGCCATTATCCTAAGTGAATTAATGCAGAAACGGA
 >chr1:89609587-89609908 - E=2e-181
AAACCAAATACTCCATGTTCTCACTTGTAAGTGGGAGCTAAACAATGAATACACATGTACATAAAGACGGGAACAATATACACTGGGGA

ATACAAGAGGGAGGAGGCAGGGAGGGAAGTAGGGTTGAAAAACGACCTTTGGGGTTCTACATTCACTATCTGGGTGACTGTTTTGACTG

AAGCCCAAACCTCAGTATCACGCAATATAGTCATGTAACAAACCTGCATGC|GAA|ATGACCATTTCTGGAGCTAGAGAAAGACAAGGA
                                                    >chr1:89726436-89726694 - E=1e-143
GGGTGAATAATTACAGAAATAGGTTAGATGCACGTGAATGGAAGAGAGATATTCAGGAACTGTTTACTGCTACTGTAGATGAAAAGAAG

TAAGGACAAGTGAGTGATTTAGGCAATCAGTAAGAAGCTTGATCTTACTTTCTATTTATCACTCTTTAGGAACAGGCTGCACAGCTCAG

CACAACATTCCAAGCTCAAAATAGAAGCCTTCTCAGTGAGCTCC
```
